# Topography-derived wetness indices are associated with household-level malaria risk in two communities in the western Kenyan highlands

**DOI:** 10.1186/1475-2875-7-40

**Published:** 2008-02-29

**Authors:** Justin M Cohen, Kacey C Ernst, Kim A Lindblade, John M Vulule, Chandy C John, Mark L Wilson

**Affiliations:** 1 Department of Epidemiology, School of Public Health, University of Michigan, Ann Arbor, MI 48109, USA; 2 National Center for Zoonotic, Vector-Borne, and Enteric Diseases, Centers for Disease Control and Prevention, Atlanta, GA 30033, USA; 3 Kenya Medical Research Institute, Kisumu, Kenya; 4 Department of Pediatrics, University of Minnesota Medical School, Minneapolis, MN 55455, USA

## Abstract

**Background:**

Transmission of *Plasmodium falciparum *generally decreases with increasing elevation, in part because lower temperature slows the development of both parasites and mosquitoes. However, other aspects of the terrain, such as the shape of the land, may affect habitat suitability for *Anopheles *breeding and thus risk of malaria transmission. Understanding these local topographic effects may permit prediction of regions at high risk of malaria within the highlands at small spatial scales.

**Methods:**

Hydrologic modelling techniques were adapted to predict the flow of water across the landscape surrounding households in two communities in the western Kenyan highlands. These surface analyses were used to generate indices describing predicted water accumulation in regions surrounding the study area. Households with and without malaria were compared for their proximity to regions of high and low predicted wetness. Predicted wetness and elevation variables were entered into bivariate and multivariate regression models to examine whether significant associations with malaria were observable at small spatial scales.

**Results:**

On average, malaria case households (n = 423) were located 280 m closer to regions with very high wetness indices than non-malaria "control" households (n = 895) (t = 10.35, p < 0.0001). Distance to high wetness indices remained an independent predictor of risk after controlling for household elevation in multivariate regression (OR = 0.93 [95% confidence interval = 0.89–0.96] for a 100 m increase in distance). For every 10 m increase in household elevation, there was a 12% decrease in the odds of the house having a malaria case (OR = 0.88 [0.85–0.90]). However, after controlling for distance to regions of high predicted wetness and the community in which the house was located, this reduction in malaria risk was not statistically significant (OR = 0.98 [0.94–1.03]).

**Conclusion:**

Proximity to terrain with high predicted water accumulation was significantly and consistently associated with increased household-level malaria incidence, even at small spatial scales with little variation in elevation variables. These results suggest that high wetness indices are not merely proxies for valley bottoms, and hydrologic flow models may prove valuable for predicting areas of high malaria risk in highland regions. Application in areas where malaria surveillance is limited could identify households at higher risk and help focus interventions.

## Background

**Figure 1:**
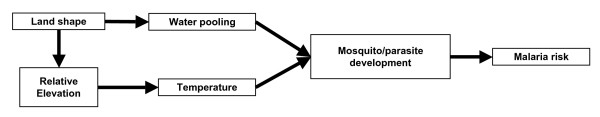
**Theoretical relationship between elevation, land-shape, and malaria**. Proposed relationship between the shape of the land, relative elevation, and malaria risk. The relationship between household elevation and malaria risk is confounded by the local shape of the land.

Elevation has long been recognized to be associated with malaria [1] due to its association with cooler temperatures [2] that slow the development of anopheline vectors and the *Plasmodium *parasites they transmit [3]. Variation in the local shape of the land also may play an important role in determining regions of suitability for mosquito breeding at smaller spatial scales (Figure 1) [4]. Malaria risk may diminish within a few hundred meters from known breeding sites [5-8], although a number of vector and environmental factors have been found to influence this range [9-12]. In highland regions of East Africa, where unstable malaria transmission may result in part from the very low numbers of anopheline mosquito vectors [13], the proximity of houses to locations with suitable topography for mosquito breeding may be an important determinant of malaria risk [9].

*Anopheles *breeding sites may occur where water collects and pools for a period of time sufficient to permit larval development and adult emergence [14]. Small temporary pools and larger more permanent ones are more likely to exist in flat, relatively low-lying regions [9]. Such areas can be identified using hydrologic techniques that model how water moves across a given surface [15]. Recently, Mushinzimana *et al *[16] used this sort of approach to demonstrate associations between modelled wetness and larval habitat in a region of western Kenya ~30 km (but hundreds of meters lower elevation) from the study site for this project.

Balls *et al *have characterized risk of malaria in a highland region of Tanzania as a broad altitudinal trend modified at smaller spatial scales by the local topography [4]. Understanding this finer-scale heterogeneity within communities is useful for two principal reasons. First, it may permit identification of high-risk regions in a community that could help focus limited intervention resources to produce maximum effect (i.e., spatial variability in risk is a characteristic of interest) [9]. Second, such heterogeneity in risk means that observed relationships between any spatially varying risk factors and pathogen transmission may be confounded unless models control for baseline risk that varies by the local topographic environment (i.e., spatial variability in risk is a nuisance obstructing investigations of other characteristics of interest). For example, the observed association between malaria risk and a truly protective household factor may be attenuated (or, in extreme cases, appear to be in the opposite direction) if that household factor occurs more frequently in a region of the community in which landscape variations result in higher baseline exposure to potentially infectious mosquitoes, and thus malaria risk, than elsewhere.

Accordingly, the association between malaria and the topographic wetness index (TWI), an approximate measure of predicted water accumulation, was assessed in two communities in the western Kenyan highlands. The TWI is calculated as the ratio of the area upslope from any given point on the landscape to the local slope at that point, and thus represents the amount of water that should enter a given spatial unit divided by the rate at which the water should flow out of that unit. The TWI is an appealing measure of the shape of the land because it provides a simple, biologically meaningful description of how topography may affect malaria risk via suitability for potential mosquito breeding. Such an index may prove useful in identification of areas within communities at higher transmission risk or in controlling for strong geographically determined baseline risk, thereby producing less-biased estimates of risk at the household level. In this study, the potential utility of the TWI in predicting risk of malaria in a highland area at small spatial scales was compared to the predictive ability of elevation alone.

## Methods

### Study site

**Figure 2:**
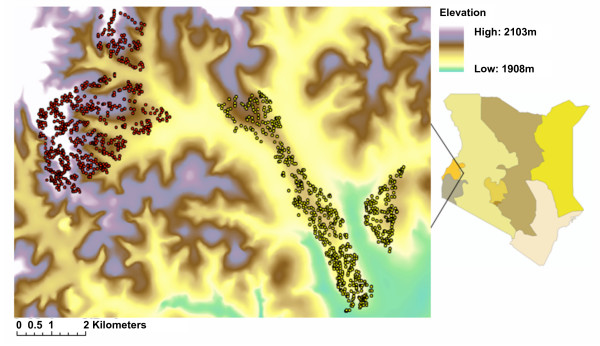
**Elevation map of Kipsamoite and Kapsisiywa**. Digital terrain map of Kipsamoite (red) and Kapsisiywa (yellow) located in Nandi District in the western Kenyan highlands. Kipsamoite elevation is higher and more variable than Kapsisiywa. Colours in the map of Kenya at right depict provinces.

This study was conducted in the Kipsamoite sublocation and Kapsisiywa location of Nandi District in the highlands of western Kenya (Figure 2). The sites are adjacent and lie between 0°16'55.64° N to 0°21'52.40° N latitude and 34°59'7.17" E to 35°5'19.90" E longitude. Elevation in Kapsisiywa ranges from 1,887 m to 1,982 m, while Kipsamoite varies between 1,950 m and 2,100 m. The principal occupations of inhabitants in both villages included subsistence (maize and some vegetables) and cash crop (tea) farming and animal husbandry (cattle, goats, sheep and chickens). Malaria incidence is highly seasonal in both communities, with heavy rains, generally between late March and early May, often followed by a peak in malaria during June or July. Epidemics of malaria have occurred in this area [17]. As in other highland areas of East Africa, the predominant vector found during entomologic evaluations was *An. gambiae *s.l. (97.5%) [18].

### Study population

All residents of Kipsamoite and Kapsisiywa were identified and assigned individual identification numbers. Births, deaths and migration were monitored quarterly during 2001–2004 in Kipsamoite and 2003–2004 in Kapsisiywa. An estimated 3,682 people lived in Kipsamoite and 3,433 in Kapsisiywa during the study period. Each house in the study region was georeferenced using a differential Trimble Pathfinder GPS system (estimated accuracy +/-1 m) and its elevation was measured. Total person-time contributed within each household was calculated separately for each year. The number of people presenting with slide-confirmed *P. falciparum *malaria at the sole health clinic in each community was tallied yearly from 2001–2004 in Kipsamoite and from April 2003 (when disease detection began in that community) to the end of 2004 in Kapsisiywa. The number of malaria events per household was calculated for each year in which data was available. Individuals presenting with malaria more than once during the same year were counted as multiple cases as long as events occurred more than 30 days apart.

### Topographic variables

A 10 m resolution Digital Terrain Model (DTM) derived from topographic maps was obtained courtesy of BIOTA Subproject E02 [19]. In this DTM, elevation across the landscape was recorded in 10 m-by-10 m grid-cells. The DTM was georeferenced and verified for internal consistency against both a 90 m resolution Digital Elevation Model (DEM) created from Shuttle Radar Topography Mission (SRTM) satellite imagery and GPS ground readings obtained using a Trimble Pathfinder unit with 1 m accuracy. The resulting georeferenced DTM was used to calculate elevation and predicted wetness across the landscape. The open source GIS program SAGIS [20] was used to compute the topographic wetness index (TWI) using Tarboton's Deterministic Infinity method [21]. Although a variety of algorithms for determining the expected flow of water over a given landscape have been developed [15], Tarboton's method was selected as a robust yet simple procedure producing reasonably realistic results [21]. The resulting TWI layer had a predicted wetness value for each of the 604,595 10 m^2 ^grid-cells across the landscape, representing the ratio of the area upslope of each 10 m^2 ^cell to the local slope for that cell. The type of land-cover or land-use occurring in each cell was evaluated by comparison with an orthorectified, 1 m pan-sharpened resolution IKONOS satellite image classified with the object-based classification program eCognition Professional 4 (Definiens AG, München, Germany).

The predicted wetness at each 10 m^2 ^grid-cell in which a household was located was obtained using ArcGIS 9.1 (ESRI, Redlands, CA) with Hawth's Analysis Tools [22]. The predicted wetness and elevation variables then were classified into ten equal-interval categories, the size of which was determined by taking the difference between maximum and minimum values of all grid-cells within 1 km of houses and dividing by ten. This classification into ten categories (where category 1 was driest or highest and category 10 wettest or lowest) was performed separately for Kipsamoite and Kapsisiywa so that the resulting variables would reflect the different wetness and elevation ranges surrounding the houses in those two communities. Distances from each house to the nearest 10 m^2 ^location in each of the ten wetness categories were calculated in ArcGIS so as to measure the proximity of houses to areas with varying suitability for the presence of standing water. Additionally, distances from each house to the nearest 10 m^2 ^location in each of the ten elevation categories were calculated to represent the proximity of homes to the lowest, highest, and in-between altitude areas of the landscape.

### Rates and case counts

The clinically-derived case data and calculated person-time measures were used to derive incidence rates over the duration of the study. The malaria incidence rate among households closer than the median distance to regions of the highest predicted wetness (category 10) was compared to that for houses farther than the median by calculating confidence intervals and a 2-sample z test assuming a normal approximation. For comparison, the rate of malaria for houses above and below the median elevation also was calculated.

To examine whether patterns of malaria surrounding very wet locations might appear analogous to those surrounding known breeding sites in other studies [5-8], houses located within 500 m of grid-cells in wetness category 9 or 10 were compared by chi-square test to those farther from such regions with respect to the number of malaria cases observed. Although published distance of risk gradients vary [9], 500 m was selected as a plausible cut-off point since sharp declines in risk are generally reported at greater distances [5-8].

### Bivariate analysis

Households in which any resident had malaria (case households) were compared to those without malaria (control households) with respect to elevation and wetness variables. Predicted wetness and elevation of the 10 m^2 ^grid-cell containing the location of each household was compared in SAS 9.1 (SAS Institute, Cary, NC) using Satterthwaite's t-test for unequal variance to calculate the statistical significance of observed differences. T-tests were also used to compare the average distances between case and control households and the nearest location in each of the ten predicted wetness and elevation categories. Analyses were performed for both communities combined, and then repeated for each individual community to examine whether the overall patterns remained consistent at those smaller spatial scales. Additionally, chi-square tests for trend were used to compare houses in Kipsamoite with malaria in multiple seasons over 2001–2004 to houses without malaria with respect to their proximity to regions of high wetness to examine the consistency of patterns over a longer time period.

### Multivariate analysis

Repeated measures multivariate logistic regression models (presence or absence of any malaria cases) and negative binomial regression models (number of malaria cases) were calculated in SAS with GENMOD, using a REPEATED statement to indicate the correlation between the two duplicate entries for each house present in both 2003 and 2004 and an exchangeable correlation structure. Modelling each year separately in this manner permitted the models to account for the specific person-time contributed each year. All models were controlled for year and household contributed person-time for that year. Variables representing the distances between houses and the nearest location in each wetness category were entered into models additionally controlling for household elevation in order to ascertain whether elevation accounted for any significant associations. Comparable variables representing the distance between houses and the nearest location in each elevation category also were entered into models controlling for household elevation to evaluate whether the temperature-mediated effect of elevation accounted for any association between household malaria and proximity to regions of relatively low elevation. Finally, in order to ascertain whether predicted wetness accounted for household malaria better than simple proximity to relatively low elevation points (which may represent valley bottoms) or absolute elevation of households, variables were entered jointly into multivariate models. As with the bivariate analysis, models were calculated using all houses in the study site together, as well as for each community separately, in order to examine the robustness of conclusions to the chosen size of the study region.

## Results

### Demography, case data, and topography

From April to December 2003, Kipsamoite had 3,823 residents living in 674 houses. During the same time period, Kapsisiywa had 3,601 residents living in 639 houses. During 2004, Kipsamoite's population increased to 3,978 residents living in 695 houses, while Kapsisiywa grew to 3,720 residents in 650 houses. Overall, 1,378 houses were occupied during the two-year period.

In Kipsamoite, 77 different individuals presented at the clinic with malaria from April through December 2003, and 120 presented during 2004. In Kapsisiywa, 246 different individuals presented at the clinic with malaria from April to December 2003, and 270 presented during 2004. Overall, 144 of 710 Kipsamoite houses (20.3%) occupied during the study had at least one case, while 281 of 668 Kapsisiywa houses (42.1%) ever occupied had at least one case. In Kipsamoite, where malaria data collection began in 2001, 93 of 565 houses (16.5%) had cases in 2001 and 136 of 603 (22.6%) had cases in 2002. During 2001 through 2004 in Kipsamoite, 467 houses (62.9%) had no cases, 196 (26.4%) had cases in only one year, 51 (6.9%) had cases in two years, 24 (3.2%) had cases in three years, and five (0.7%) had cases in all four years.

**Figure 3:**
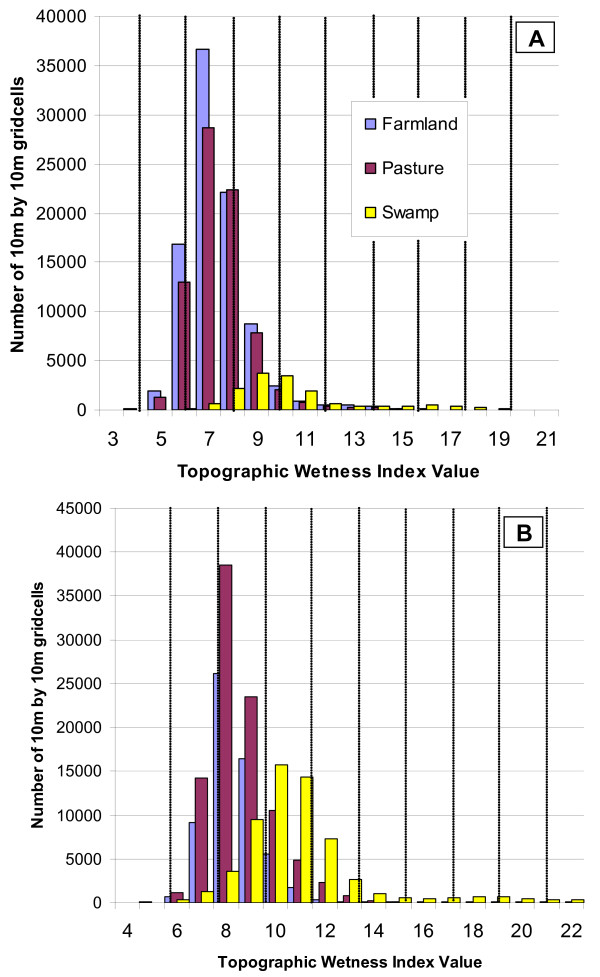
**Predicted wetness of categorized land-cover**. The predicted wetness of farmland, pasture, and swamp within (a) Kipsamoite and (b) Kapsisiywa, represented by the number of 10 m^2 ^grid-cells. Vertical lines mark the divisions between each of the ten wetness categories used in analysis.

The TWI values, representing predicted wetness, averaged 7.7 (range: 2.9 to 21.8) within 1 km of houses in Kipsamoite (Figure 3). Within 1 km of houses in Kapsisiywa, the TWI averaged 9.0 (range: 4.4 to 23.4). In Kipsamoite, farmland in non-swamp regions had an average of 7.4, compared to 8.4 in Kapsisiywa. Non-farmed pasture had a similar TWI average of 7.5 in Kipsamoite and 8.7 in Kapsisiywa. TWI in regions within swamp margins averaged 10.4 in Kipsamoite and 10.9 in Kapsisiywa.

### Rates and case counts

Across the entire study site, houses located closer than the median distance to areas of highest predicted wetness had a malaria incidence rate of 91.2 cases/1000 person-years (py) (95% CI = 83.8 – 98.7), ~3 times greater (z = 13.7, p < 0.001) than houses farther than the median (30.5 cases/1000 py) (26.2 – 34.9). Joint comparison of proximity to wetness and household elevation in a 2-by-2 table demonstrated increased malaria rates among houses located closer than the median to regions of high predicted wetness regardless of elevation (Table 1). Among houses above the median elevation, those closer to regions of highest predicted wetness had 44.9 more cases/1000 py than those located farther away (z = 7.93, p < 0.001). Among houses below median elevation, those closer to highest predicted wetness had 47.6 more cases/1000 py than those farther away (z = 5.03, p < 0.001). The effect of elevation appeared to be smaller but still statistically significant, since houses at lower elevation had only 24.1 more cases/1000 py than those at higher elevation (z = 4.31, p < 0.001) when located far from very wet regions, and had 26.8 more cases/1000 py when close to very wet regions (z = 2.97, p = 0.001).

**Table 1:** Rates of malaria by elevation and proximity to predicted wetness

		**Household elevation**	
		
		**Above median**	**Below median**
**Distance to wettest locations**	**Above median**	25.7 (21.2–30.2) RR = Reference	49.8 (37.3–62.3) RR = 1.93
	**Below median**	70.6 (57.0–84.3) RR = 2.75	97.4 (88.6–106.2) RR = 3.74

Malaria rates per 1000 person-years (95% confidence intervals) and relative rates (RR) during 2003–2004 for houses located higher and lower than the median elevation for their community and closer or farther than the median from locations of highest predicted wetness.

Houses ≤ 500 m from the two wettest categories (9 and 10) were significantly more likely to have malaria cases than houses >500 m from such regions (χ^2 ^= 3.89, p < 0.001). Of houses with ≤ 3 cases, 39.5% (34/86) were <500 m from a very wet region, compared to 26.7% (24/90) of houses with 2 cases, 22.0% (54/246) of houses with 1 case, and 14.0% (125/894) of houses with no cases in either year.

### Bivariate and multivariate associations for predicted wetness distances

Case households were situated in grid-cells with higher average predicted wetness 7.79 (SD = 0.88) than control households 7.46 (SD = 1.05) (Satterthwaite t = -5.95, 977 df, p < 0.0001). However, case and control households did not differ significantly in their average predicted wetness at the location of households when separately considering Kipsamoite (t = -1.91, 261 df, p = 0.57) or Kapsisiywa (t = 0.76, 631 df, p = 0.45).

In each community separately, households with malaria cases in either 2003 or 2004 were located significantly and progressively closer to landscape classified into the highest predicted wetness categories. In Kipsamoite, case households were on average 40.9 m closer to category 7 (t = 2.33, 237 df, p = 0.021), 64.9 m closer to category 8 (t = 2.92, 224 df, p = 0.004), 110.8 m closer to category 9 (t = 4.11, 226 df, p < 0.001), and 223.6 m closer to category 10 (t = 4.45, 203 df, p < 0.001) than were households without cases in 2003 or 2004. These case households were located significantly farther from regions in category 1 (lowest wetness) than were control households. In Kapsisiywa, households with cases in either 2003 or 2004 were located an average 84.0 m closer to landscape classified into the highest predicted wetness category 10 (t = 3.95, 638 df, p < 0.001).

When these same distance variables were entered into multivariate models that controlled for year and person-time, statistically significant associations remained between household malaria and distance to each of the three highest predicted wetness categories. Associations remained statistically significant after additionally controlling for community, and no interactions between distance to wetness variables and community were significant (data not shown).

Each community also was examined separately to ascertain whether results were robust to the smaller spatial scale and sample size. In Kipsamoite, distance to the four highest wetness categories remained significantly associated with malaria households (Table 2). Similarly, in Kapsisiywa, distances to the nearest locations in each of the three wettest categories were significantly associated with the presence of household malaria (Table 2). After additionally controlling for household elevation, proximity to a region in the two wettest categories remained a significant predictor of disease in both Kipsamoite and Kapsisiywa. Negative binomial models using the case count for each season as an outcome produced very similar results (data not shown).

**Table 2:** Malaria odds ratios associated with distance to wetness categories

		**Controlling for person-time and year**			**Controlling for person-time, year, and elevation**		
		
**Wetness category**	**Wetness values**	**OR**	**95% CI**		**OR**	**95% CI**	
		*for a 100 m increase in distance*			*for a 100 m increase in distance*		
Kipsamoite							

1 (driest)	2.9–4.8	**1.084**	1.046	1.123	**1.065**	1.005	1.129
2	4.8–6.7	1.078	0.879	1.322	0.812	0.610	1.079
3	6.7–8.6	**0.204**	0.060	0.695	**0.295**	0.089	0.974
4	8.6–10.5	1.031	0.810	1.312	1.253	0.938	1.674
5	10.5–12.4	1.033	0.868	1.230	1.103	0.923	1.318
6	12.4–14.3	0.940	0.818	1.080	1.066	0.915	1.243
7	14.3–16.2	**0.894**	0.817	0.978	0.993	0.874	1.128
8	16.2–18.1	**0.882**	0.815	0.953	0.924	0.841	1.016
9	18.1–20.0	**0.870**	0.812	0.934	**0.892**	0.827	0.963
10 (wettest)	20.0–21.8	**0.920**	0.885	0.957	**0.932**	0.894	0.972

Kapsisiywa							

1 (driest)	4.4–6.3	0.987	0.902	1.080	0.971	0.883	1.067
2	6.3–8.2	0.749	0.515	1.089	0.684	0.465	1.006
3	8.2–10.1	1.070	0.752	1.524	1.106	0.776	1.575
4	10.1–12.0	1.003	0.895	1.125	1.027	0.905	1.165
5	12.0–13.9	0.972	0.896	1.055	0.982	0.899	1.072
6	13.9–15.8	0.932	0.858	1.011	0.936	0.860	1.019
7	15.8–17.7	0.929	0.862	1.002	0.933	0.863	1.009
8	17.7–19.6	**0.924**	0.856	0.997	0.927	0.853	1.007
9	19.6–21.5	**0.917**	0.854	0.984	**0.909**	0.838	0.987
10 (wettest)	21.5–23.4	**0.903**	0.857	0.953	**0.894**	0.841	0.950

Odds ratios for the presence of any household malaria in 2003–2004 associated with 100 m increases in distance between houses and the nearest 10 m^2 ^grid-cell in each of ten wetness categories.

**Figure 4:**
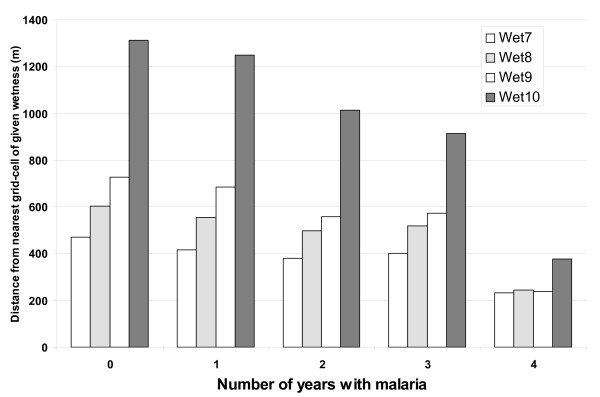
**Predicted wetness for houses with recurrent malaria**. Distribution of distances to the nearest cell in the four highest wetness categories for Kipsamoite houses with malaria cases in 0, 1, 2, 3, or 4 years. Increasing numbers of years are significantly associated with closer proximity to all four categories.

In Kipsamoite during 2001–2004, houses with no malaria (n = 401), or malaria during any one (n = 192), two (n = 51), three (n = 24), or all four (n = 5) years, were compared with respect to their proximity to the nearest location in the four highest predicted wetness categories to evaluate consistency over time (Figure 4). Each additional season that a house had at least one malaria case was associated with an average 139.8 m closer to a region of highest (category 10) predicted wetness (χ^2 ^= 37.38, p < 0.0001), 69.7 m to the second wettest (category 9) (χ^2 ^= 27.15, p < 0.0001), 48.4 m to the third wettest (category 8) (χ^2 ^= 18.62, p < 0.0001), and 40.1 m to the fourth wettest (category 7) (χ^2 ^= 18.79, p < 0.0001).

### Bivariate and multivariate associations for elevation

**Figure 5:**
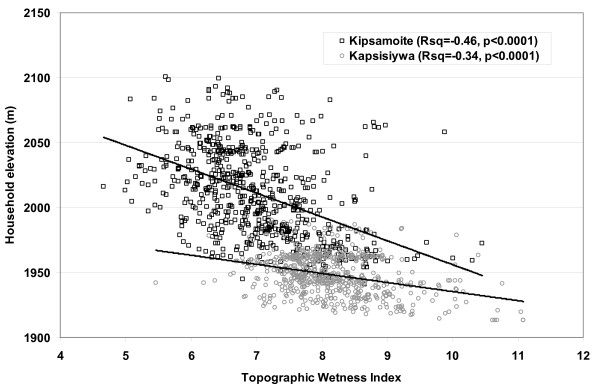
**Relationship between elevation and predicted wetness**. Plot of predicted wetness (X-axis) at the location of households in Kipsamoite (black squares) and Kapsisiywa (gray circles) against household elevation (Y-axis). The overall r = -0.66 compared to -0.46 for Kipsamoite and -0.34 for Kapsisiywa.

Predicted wetness was significantly correlated with elevation at the location of households (r = -0.66; p < 0.0001). Community-specific correlations also were statistically significant for both Kipsamoite (r = -0.46; p < 0.0001) and Kapsisiywa (r = -0.34; p < 0.0001) (Figure 5). In Kipsamoite, households with cases in 2003 or 2004 were located at an average elevation of 2,003.4 m (SD = 35.8) compared to an average of 2,013.5 m (SD = 34.7) for controls, a statistically significant difference (t = 3.01, 219 df, p = 0.003). In Kapsisiywa, however, there was no significant difference (t = -1.07, 607 df, p = 0.27) between average elevation of cases (1,949.0 m, SD = 15.6) and controls (1,947.6 m, SD = 16.0).

Household elevation was entered into multivariate logistic regression models to control for year and contributed person-time at risk. Overall, a 10 m increase in elevation was associated with a household malaria odds ratio (OR) of 0.88 (0.85–0.90), indicating a 12% decrease in odds of malaria. After stratifying by community, this relationship remained significant for Kipsamoite (OR = 0.92 [0.87–0.97]) but not for Kapsisiywa (OR = 0.96 [0.89–1.05]).

Distance between houses and the nearest location in various elevation categories also was associated with malaria. In Kipsamoite, case households were significantly closer to areas within the three lowest elevation categories: 1,965–1,982 m (t = 2.96, 225 df, p = 0.003), 1,948–1,965 m (t = 2.98, 226 df, p = 0.003), and 1,931–1,948 m (t = 3.09, 219 df, p = 0.002). In Kapsisiywa, however, case households were significantly farther from areas in the lowest two elevation categories: 1,920–1,930 m (t = -2.08, 643 df, p = 0.025) and 1,911–1,920 m (t = -2.21, 560 df, p = 0.028). After controlling for person-time and year, closer proximity to regions of very low elevation was associated with increased risk of household malaria in Kipsamoite, but not in Kapsisiywa (Table 3).

**Table 3:** Malaria odds ratios associated with distance to elevation categories

		**Controlling for person-time and year**			**Controlling for person-time, year, and elevation**		
**Elevation category**	**Elevation values**	**OR**	**95% CI**		**OR**	**95% CI**	
		*for a 100 m increase in distance*			*for a 100 m increase in distance*		

Kipsamoite							

1 (highest)	2085–2102	**1.022**	1.004	1.040	1.001	0.978	1.024
2	2068–2085	1.020	1.000	1.040	0.991	0.964	1.018
3	2051–2068	**1.042**	1.020	1.063	1.030	0.994	1.067
4	2034–2051	**1.051**	1.029	1.073	**1.048**	1.010	1.087
5	2017–2034	**1.078**	1.042	1.117	**1.058**	1.003	1.115
6	2000–2017	**1.061**	1.005	1.119	1.013	0.950	1.081
7	1982–2000	0.962	0.870	1.064	0.979	0.900	1.066
8	1965–1982	**0.855**	0.776	0.943	0.919	0.781	1.080
9	1948–1965	**0.905**	0.851	0.962	0.949	0.837	1.077
10 (lowest)	1931–1948	**0.957**	0.934	0.981	0.976	0.932	1.022

Kapsisiywa							

1 (highest)	1996–2005	0.995	0.989	1.002	**0.985**	0.975	0.994
2	1986–1996	0.995	0.988	1.002	**0.984**	0.974	0.994
3	1977–1986	**1.014**	1.002	1.025	**1.018**	1.003	1.034
4	1967–1977	1.012	0.992	1.032	1.011	0.984	1.040
5	1958–1967	1.005	0.978	1.033	0.994	0.958	1.032
6	1949–1958	1.001	0.970	1.034	0.991	0.954	1.029
7	1939–1949	0.944	0.871	1.023	0.949	0.876	1.028
8	1930–1939	0.953	0.879	1.033	0.959	0.861	1.069
9	1920–1930	0.999	0.984	1.014	1.007	0.987	1.028
10 (lowest)	1911–1920	1.000	0.989	1.012	1.008	0.992	1.024

Odds ratios for the presence of any household malaria in 2003–2004 associated with 100 m increases in distance between houses and the nearest 10 m^2 ^grid-cell in each of ten elevation categories.

### Comparison of predicted wetness and elevation

Distances from houses to the nearest grid-cells in the two highest predicted wetness categories were consistently associated with malaria in multivariate regression models for the entire study site, as well as individually for Kipsamoite and Kapsisiywa, even after controlling for elevation variables (Table 4). Interactions between predicted wetness and elevation variables were not significant in these models.

**Table 4:** Multivariate regression with predicted wetness and elevation variables

		**Kipsamoite**			**Kapsisiywa**		
**Type of model**	**Variables in model**	**OR**	**95% CI**		**OR**	**95% CI**	

Predicted wetness or elevation variables alone	Distance to very high predicted wetness	**0.920**	**0.885**	**0.957**	**0.903**	**0.857**	**0.953**
	Household elevation	**0.422**	**0.251**	**0.710**	0.677	0.293	1.564
	Distance to very low elevation	**0.958**	**0.935**	**0.982**	1.009	0.999	1.019

Predicted wetness and elevation variables jointly	Distance to very high predicted wetness	**0.925**	**0.888**	**0.964**	**0.874**	**0.826**	**0.925**
	Household elevation	0.686	0.401	1.174	**3.552**	**1.475**	**8.550**
	Distance to very high predicted wetness	**0.923**	**0.884**	**0.963**	**0.897**	**0.854**	**0.942**
	Distance to very low elevation	0.985	0.960	1.012	**1.012**	**1.002**	**1.023**

Odds ratios for the presence or absence of household malaria associated with a 100 m increase in distance or elevation, entering variables singly and jointly into multivariate logistic regression models. Models control for year and person-time.

Household elevation was a significant negative predictor of disease overall, even after controlling for predicted wetness variables (for example, when controlling for distance to category 10 wetness, year, and person-time, each 10 m increase in household elevation was associated with OR = 0.93 [0.90–0.97]), but was not consistently associated with disease when controlling for community (OR = 0.98 [0.94–1.03]). Although household elevation and the distance between houses and grid-cells in low elevation categories in Kipsamoite were associated with disease when controlling for year and person-time (for example, a 100 m increase in distance to lowest elevation category 10 was associated with OR = 0.96 [0.93–0.98]) these variables were not independent predictors of household malaria when additionally controlling for variables representing the distance between houses and grid-cells of very high predicted wetness (OR = 0.98 [0.96–1.01]). In Kapsisiywa, elevation variables were not significantly associated with household malaria when controlling for year and person-time (p > 0.1 for all), and were positively associated with malaria when entered jointly with distance to high predicted wetness (i.e., higher elevation and increased distance from very low points were both associated with increased risk of household malaria when controlling for distance to high wetness).

## Discussion

The varied topography of highland regions and its likely effects on malaria transmission underscore the need to consider not only household- and individual-level factors, but also broader geographic and environmental contextual determinants of risk [23]. Although general topographic trends have been described in relation to increased transmission [13] and vector densities [24] at lower altitudes or within valley bottoms [25], simple methods for predicting regions of risk are lacking. Such a means of identifying regions of a community at particularly high risk of malaria transmission would permit the focusing of limited intervention resources for maximum effectiveness [9]. In addition, a simple means of characterizing heterogeneities in transmission risk related to the landscape in which people live could be used to control for spatial confounding of associations between malaria and other spatially-varying determinants of interest, such as household risk factors. In this study, associations between household malaria and one possible risk measure, the topographic wetness index, were investigated. Results indicate the TWI, which here is derived solely from elevation, may describe malarial risk at small spatial scales even when insufficient variation exists in local elevation for that variable to manifest meaningful associations with household malaria.

As expected, there was a strong correlation between elevation and predicted wetness, and both variables were associated with malaria at the household level across the study site. However, when considering only the smaller spatial scales and lesser variance occurring within the individual communities, elevation was found to be significantly associated with disease in Kipsamoite (the community with higher and more variable elevation) but not in Kapsisiywa, where the variation in household elevation was insufficient to demonstrate meaningful associations. In Kipsamoite, variables related to elevation were not independent predictors of risk in models that included proximity to regions of highest predicted wetness, while in Kapsisiywa, the same elevation variables were inversely associated with household malaria (i.e. higher elevation was associated with more malaria risk) (Table 4). As such, elevation appeared to determine the general trend in malaria incidence across the entire study site (for example, houses in Kapsisiywa were located at lower elevation than those in Kipsamoite, and subsequently had higher incidence), but it was less useful for prediction of households with disease when restricting to intra-community spatial scales where little altitudinal variation occurred.

The relationship between high predicted wetness and household malaria was consistent both across the study site and also robust to the smaller spatial scales of the individual communities, even when insufficient variation in elevation existed to manifest statistical associations with malaria. In multivariate analysis, distance to regions of very high wetness remained a significant predictor of risk while distance to very low elevation points did not, suggesting that a high TWI value is not merely a proxy for a valley bottom. Although the two measures were correlated across the study site (r = -0.66 at the location of households), considerable variation existed between predicted wetness and elevation at an intra-community scale (Figure 5). Evaluation of the four years of case data from Kipsamoite revealed a consistent association between malaria and proximity to high predicted wetness over the time period (Figure 4). Those houses in which individuals developed clinical malaria in multiple years were located much closer to regions of high predicted wetness than were houses without malaria. Previous research at this study site identified persistent spatial clusters of malaria transmission that may be consistent with this finding [18].

The topographic wetness index presented here is a simple measure, and it did not incorporate factors such as heterogeneity of rainfall, soil characteristics, vegetation differences, or human modification of the environment, each of which may affect the collection of water in a given region. Addition of such variables may improve the prediction of these models. Nevertheless, a body of hydrological and geographical literature supports the use of such a simple index in prediction of surface water availability [26]. It is possible that in other regions with greater variation in climatic and environmental conditions, incorporation of such heterogeneities into wetness calculations may be essential for proper hydrologic predictions.

Predicted wetness in the 10 m^2 ^grid-cell containing each household location was not a statistically significant predictor of household-level malaria when stratifying by community, but houses were generally located in relatively dry locations, with an average distance of 580 m (SD = 451) from the swampy regions in which the very wet locations identified here were located. It is well known that malaria vectors may travel some distance to find a blood meal [9], though studies of malaria risk around known breeding sites, including swamps, have demonstrated increased transmission within several hundred meters of these sites [11]. In our study, houses with multiple malaria cases were more likely to have a region of very high predicted wetness located within 500 m, providing evidence that high values of the TWI represent regions likely to allow *Anopheles *breeding habitats. Although no larval mosquito sampling was conducted to confirm these predictions, other nearby investigations have demonstrated associations between such local topography, areas of water collection, and larval habitat [16,27]. Proximity to known mosquito breeding habitat consistently has been demonstrated to be a risk factor for malaria at the household level [6,11,28]. Alternatively, recent mathematical models have demonstrated that proximity to water bodies may increase malaria risk regardless of whether those bodies are suitable for adult mosquito emergence [29], suggesting the importance of identifying potential water collection sites irrespective of larval sampling. Additional research on the movement of adult vectors would help to elucidate the etiology of these variations in malaria risk.

Most regions of very high predicted wetness identified in this study (i.e., TWI of ~18 in Kipsamoite or ~19 in Kapsisiywa) occurred within "swampy" habitats (Figure 3). Prior investigations have confirmed the importance of swamps [27,30] and valley bottoms [25] as potential habitats for mosquito reproduction, and hence foci of malaria risk in similar highland regions. Although this study did not seek explicitly to compare the predictive ability of the TWI versus proximity to "swamp," the very wet TWI regions that were consistently implicated as high malaria risk comprised only about 2.2% of the "swampy" habitats near homes. Thus, it seems likely that proximity to an area of high TWI represents a more accurate measure than proximity to swamps in general.

These results indicate that at small spatial scales, such as within individual communities, a lack of variation in elevation may prevent observation of associations with malaria incidence, despite overarching altitudinal trends at larger spatial scales. Nevertheless, measures of the shape of the land directly derived from elevation, such as the topographic wetness index, may serve an important role in predicting households at greatest malaria risk at such a scale. These findings confirm the importance of considering not only general trends in elevation when predicting regions of higher malaria risk, but also the variance around those trends attributable to local heterogeneities in the shape of the land. Local variations in land-shape may play an important role in shaping patterns of transmission [4]. It seems likely that at broader spatial scales, such topographic "noise" may prove much less important to characterizing regions at risk than will the general altitudinal trend.

## Conclusion

Elevation has important effects on transmission through its effect on temperature, but this study indicates that, at least in this highland setting, proximity of houses to pooled, standing water may provide a more useful measure of transmission risk when little variation exists in household elevation. Other characteristics of the local environment, such as land-cover and agricultural practices, may also influence or modify the patterns described here. Additionally, regions with variance in elevation dissimilar from that observed here could manifest different relationships. Verifying the consistency of these results will require comparison of malaria transmission in other highland communities with different topographies and human ecologies.

## Authors' contributions

JMC was responsible for conceptualization, environmental data gathering, data analysis and interpretation, drafting and revision of the manuscript. KCE and KAL were involved in data collection, interpretation of results, and critical revision of the manuscript. JV provided institutional support for this study and reviewed the manuscript. CCJ was involved in interpretation of results and critical revision of the manuscript. MLW participated in conceptualization, analysis, interpretation and critical revision of the manuscript. All authors read and approved the final version.
